# Clinical Features and Surgical Treatment of Primary Pulmonary Lymphoma: A Retrospective Study

**DOI:** 10.3389/fonc.2022.779395

**Published:** 2022-02-03

**Authors:** Hui Shen, Yaodong Zhou

**Affiliations:** Department of Thoracic Surgery, Fudan University Shanghai Cancer Center, Shanghai, China

**Keywords:** biopsy, pathology, lung resection, primary pulmonary lymphoma, prognosis

## Abstract

**Background:**

Primary pulmonary lymphoma (PPL) is a rare clonal lymphoproliferative lung disease. The present study analyzes the clinical features, imaging data, pathologic characteristics, treatment, and prognosis of PPL patients, with the aim to discuss the appropriate diagnosis and therapy of PPL patients in thoracic surgery.

**Methods:**

We performed a retrospective analysis on 36 patients with PPL confirmed by postoperative pathology between 2006 and 2020. We divided the patients into low-stage (IE) and high-stage (IIE) groups using modified Ann Arbor staging. The clinical manifestations, imaging findings, treatment modalities, and outcomes were evaluated.

**Results:**

The female to male ratio was 1.57:1 and the median age was 55 (31–69) years old. The majority of the patients had stage IE disease (75%; 27 of 36) and 9 patients had stage IIE disease. Patients with advancing stage were more likely to have respiratory symptoms. The imaging findings presented solid nodule or mass, pneumonia-like consolidative pattern, ground-glass opacity, and mixed pattern. There were 31 cases of mucosa-associated lymphoid tissue lymphoma (MALT), 2 diffuse large B-cell lymphoma (DLBCL), 2 nodular sclerosing Hodgkin’s lymphoma, and 1 marginal zone B-cell lymphoma. Two patients were diagnosed with PPL and non-small cell lung cancer (NSCLC) synchronously (one AIS and MIS and one lung adenocarcinoma). All the patients received surgery. Nine patients received adjuvant therapy after surgery (five radiotherapy, two chemotherapy, and two chemoradiotherapy). Thirty-four patients had a median follow-up time of 31 months (follow-up range: 7–152 months). Of the 34 patients, 1 patient died of liver metastases and 1 patient died of intestinal metastases.

**Conclusions:**

Our retrospective analysis suggested that most PPLs were indolent and had favorable prognosis, but the discrimination of PPL with other lung diseases was difficult. Preoperative biopsy and intraoperative frozen section examination might help in the surgical choice. Limited lung resection was enough for peripherally localized PPL.

## Background

Lung cancer is the most frequent cancer and the leading cause of cancer death in the world (1). Different from non-small cell lung cancer (NSCLC) and small cell lung cancer (SCLC), primary pulmonary lymphoma (PPL) is a rare clonal lymphoproliferative lung disease with no detectable extrapulmonary involvement at primary diagnosis or the subsequent 3 months, accounting for about 3.6% of extranodal lymphoma and 0.3% of primary pulmonary malignancies (2, 3). PPL can be classified into Hodgkin’s lymphoma (HL) and non-Hodgkin’s lymphoma (NHL) (4). Mucosa-associated lymphoid tissue lymphoma (MALT), B-cell lymphoma, diffuse large B-cell lymphoma (DLBCL), and T/NK-cell lymphoma are the main types of NHL (5).

It is reported that MALT is the most common subtype of PPL, accounting for up to 90% of all primary cases (6, 7). Because of its rarity, the clinical presentation and prognosis of PPL are little known. Patients with PPL often have non-specific symptoms such as cough, fever, chest pain, and dyspnea (8, 9). Most PPLs are indolent in nature and have good long-term survival (10). With the lack of a large cohort study, the Ann Arbor staging system which is developed for Hodgkin’s lymphoma is extensively used for clinical staging of PPL (11). The radiographic features of PPL vary from masses, nodules, and consolidation to ground-glass opacity (GGO) (12). As a result, PPL is easily misdiagnosed as other lung diseases, like lung cancer, pneumonia, or tuberculosis. In consideration of the indolent characteristic of PPL, many experts suggest that observation and regular follow-up are the first choice for PPL patients. Besides observation, radiotherapy and chemotherapy can also be used. It is conflicting whether PPL should make surgical resection or not. To improve our understanding of PPL, we conducted this retrospective study. In this study, we retrospectively analyzed 36 patients with primary pulmonary lymphoma treated at the Department of Thoracic Surgery of Shanghai Cancer Center to investigate the clinical features, surgical treatment, and prognosis of PPL.

## Materials and Methods

### Patient Selection

From 2006 to 2020, we retrospectively included 36 patients with primary pulmonary lymphoma from the Department of Thoracic Surgery of Shanghai Cancer Center. The inclusion criteria were as follows: 1) patients initially diagnosed at the Department of Thoracic Surgery, 2) clear diagnosis based on the postoperative pathology, 3) pulmonary (unilaterally or bilaterally) with or without hilar or mediastinal involvement, 4) no evidence of extrathoracic and extranodal tissue invasion at primary diagnosis or the subsequent 3 months, and 5) patients with surgical resection or biopsy.

### Data Collection

Medical records concerning the demographic and clinical characteristics of all patients were obtained. All patients underwent a contrast-enhanced computed tomography (CT) scan before surgery. Two radiologists were required to review the same CT data. If they disagreed with each other, a third radiologist would make a decision. In consideration of the high costs of PET-CT, not all patients received PET-CT. Instead, patients would receive radionuclide bone scan and ultrasound of the neck and abdomen. Some patients underwent pathologic evaluation by CT-guided percutaneous lung biopsy or bronchoscopy before surgery. The postoperative pathology was also reviewed by two pathologists. If pathologists could not get a clear diagnosis by routine paraffin pathology, gene rearrangement analysis was required. All patients received surgical resection or biopsy from the Department of Thoracic Surgery. Some patients received chemotherapy, radiotherapy, or chemoradiotherapy after surgery according to the histological type and tumor stages obtained by surgery. The research was approved by the Research Ethics Committee of Shanghai Cancer Center of Fudan University. All patients consented to participate in this research.

### Diagnostic and Therapeutic Approach

The preoperative diagnosis of MALT depends on the medical history, physical examination, and radiologic imaging like PET-CT, contrast-enhanced CT scan, and CT-guided percutaneous lung biopsy or bronchoscopic biopsy. If the preoperative transbronchial or CT-guided biopsy does not show malignancy, wedge resection would be performed for the suspicious nodules at first. Moreover, the intraoperative frozen section could help decide whether a more extensive procedure should be performed. The postoperative pathology was evaluated by H&E (hematoxylin–eosin staining) and immunohistochemical staining in all the patients. The interstitial infiltrate of small lymphocytes forming a mass-like lesion in the bronchiolar mucosa was the distinct morphology with the unique immunohistochemical staining of CD20(+), CD5(−), and CD10(−). When immunohistochemical staining was not sufficient for diagnosis, Ig gene rearrangement was required for further verification. In general, stage IE or IIE pulmonary MALT lymphoma with unilateral pulmonary involvement was treated only by surgical resection if the tumor was resectable. When PPL involved bilateral pulmonary or a complete resection could not be performed, adjuvant therapy was suggested. Usually, segmentectomy and lobectomy were often combined with lymph node dissection if the mediastinal lymph nodes were completely resectable.

### Statistical Analysis

Continuous data were analyzed using Student’s *t*-test or Mann–Whitney *U* test, while categorical data were analyzed using chi-square test. The survival curves of overall survival (OS) and relapse-free survival (RFS) were calculated by Kaplan–Meier survival analysis and log-rank test. Univariate and multivariate Cox regression analyses were performed to estimate the potential prognostic factors. Statistical analysis was performed using SPSS 23. *P*-value less than 0.05 was considered to be statistically significant.

## Results

### General Characteristics

In this study, we retrospectively reviewed patients in our department with histologically proven PPL diagnosed from January 2006 to November 2020. Finally, 36 patients who met the inclusion criteria were included in this study. According to the modified Ann Arbor staging system (13), the 36 patients were classified into two groups: low-stage (stage IE, *n* = 27, 75%) and high-stage (stage IIE, *n* = 9, 25%). In the high-stage patients, there were five cases of stage II 2E, one cases of stage II 1E, and three cases of stage II 2EW. The baseline characteristics of the 36 patients with PPL are summarized in **Table 1**. Of the 36 patients, there were 14 men and 22 women with a median age of 55 (31–69) years. Twenty-two patients were accidentally diagnosed by routine radiographic examination with no clinical symptoms, but 14 other patients had respiratory symptoms like cough (*n* = 7, 50%), fever (*n* = 6, 42.9%), chest distress (*n* = 4, 28.6%), chest pain (*n* = 1, 7.1%), and bloody sputum (*n* = 1, 7.1%). Twenty-one patients received antibiotics for 1 to 4 weeks before hospitalization. Only seven patients had a smoking history. As shown by the results in **Table 1**, there was no significant difference between tumor stage and clinical characteristics.

**Table 1:** Clinical characteristics of the patients with primary pulmonary lymphoma.

Characteristic	Total (*n* = 36)	Stage I (*n* = 27)	Stage II (*n* = 9)	*P*-value
Age (range)	55 (31–69)	54 (31–69)	61 (44–66)	0.087
≤60 years	25 (69.4)	21 (77.8)	4 (44.4)	0.096
Male sex	14 (38.9)	10 (37.0)	4 (44.4)	0.712
Never smoker	29 (80.6)	22 (81.5)	7 (77.8)	1.000
Comorbidities				
Hypertension	5 (13.9)	4 (14.8)	1 (11.1)	1.000
Diabetes mellitus	2 (5.6)	2 (7.4)	0 (0)	1.000
Respiratory symptoms				
Cough	7 (19.4)	3 (11.1)	4 (44.4)	0.050
Fever	6 (16.7)	4 (14.8)	2 (22.2)	0.627
Chest distress	4 (11.1)	2 (7.4)	2 (22.2)	0.255
Chest pain	1 (2.8)	0 (0)	1 (11.1)	0.206
Bloody sputum	1 (2.8)	1 (3.7)	0 (0)	1.000
Radiologic findings				0.169
Solid nodule or mass	19 (52.8)	14 (38.9)	5 (13.9)	
Pneumonia-like consolidative pattern	8 (22.2)	4 (11.1)	4 (11.1)	
Ground-glass opacity	7 (19.4)	7 (19.4)	0 (0)	
Mixed pattern	2 (5.6)	2 (5.6)	0 (0)	
IPI score				0.250
0–1	35 (97.2)	27 (100.0)	8 (88.9)	
2–3	1 (2.8)	0 (0)	1 (11.1)	
LDH				0.443
Normal	34 (94.4)	26 (96.3)	8 (88.9)	
Elevated	2 (5.6)	1 (3.7)	1 (11.1)	

### Lung Lesion Features in Radiographic Images and Preoperative Biopsy

**Figure 1:**
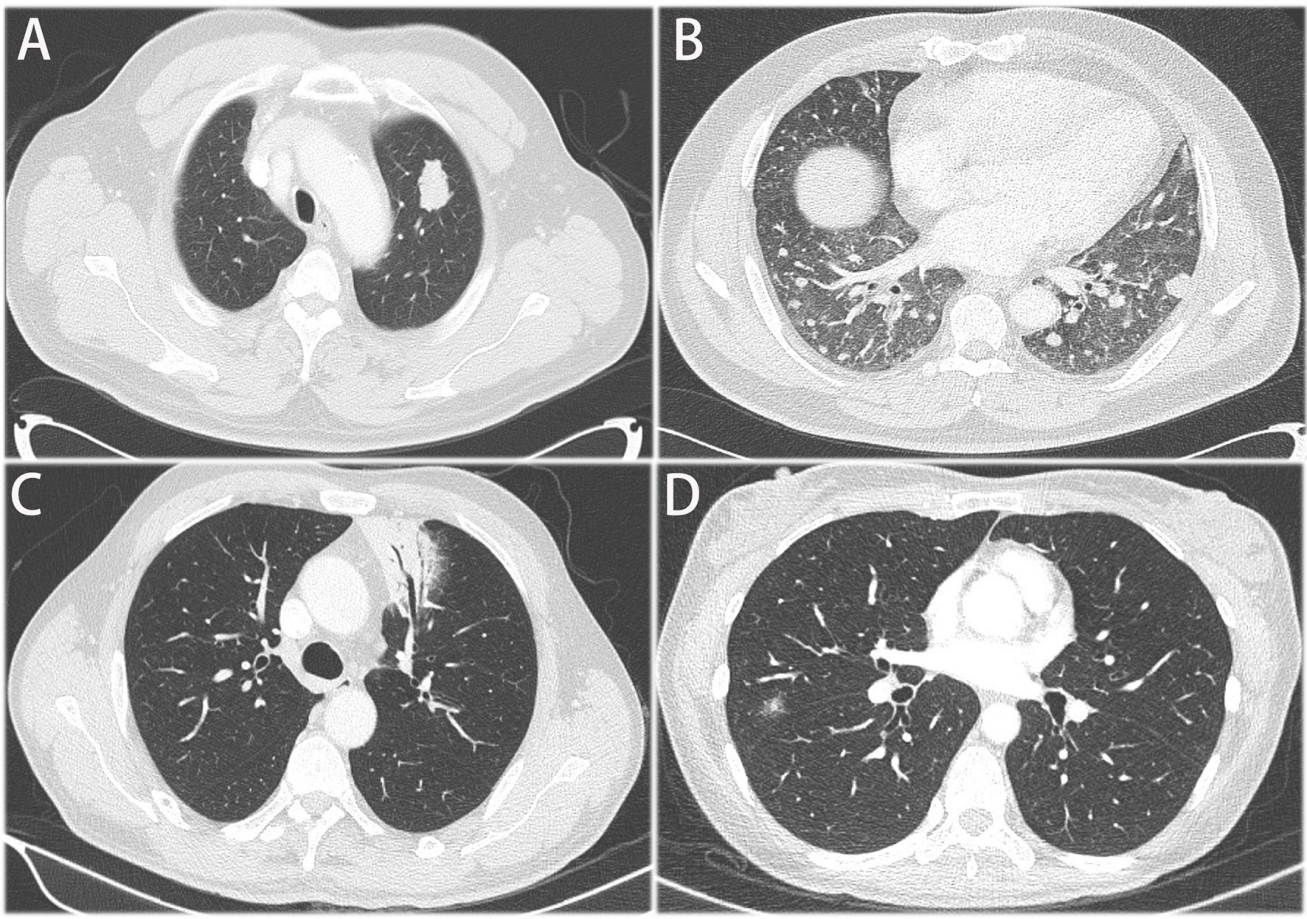
Representative CT images of primary pulmonary. **(A)** solid nodule or mass, **(B)** multiple nodules or masses **(C)** pneumonia-like consolidative pattern, **(D)** ground glass opacity.

Fourteen patients received PET-CT before surgery. Of the 14 patients, 9 patients had data of SUVmax absorption (1 patient with no absorption, 1 patient with 17.3 SUVmax, the other 7 patients with less than 6 SUVmax). All the 36 patients had contrast-enhanced CT scan before surgery. The main features could be divided into four groups: I) solid nodule or mass, II) pneumonia-like consolidative pattern, III) ground-glass opacity, and IV) mixed pattern (**Figure 1**). Most of the CT images of PPL featured a solid nodule or mass. As shown in **Table 1**, there was no significant difference between tumor stage and radiologic findings. There were seven patients with GGO featured in the CT images, which should be distinguished from early-stage NSCLC.

Thirteen patients had preoperative pathology by CT-guided percutaneous lung biopsy (*n* = 10) or bronchoscopy (*n* = 3). Of the 10 patients with percutaneous lung biopsy, 1 patient got a cytological pathology of NSCLC, but the paraffin pathology of the surgical specimen was DLBCL. Of the three patients with bronchoscopic biopsy, one patient got a cytological pathology of chronic mucositis and inflammatory cells in the stroma. Compared with bronchoscopic biopsy, percutaneous lung biopsy was much more accurate, but still could not be fully trusted. Paraffin pathology of the surgical specimens is still the golden standard. Radiographic images like PET-CT or CT and preoperative pathology could help assist with clinical diagnosis.

### Surgical Options, Pathology Characteristics, and Prognosis

**Figure 2:**
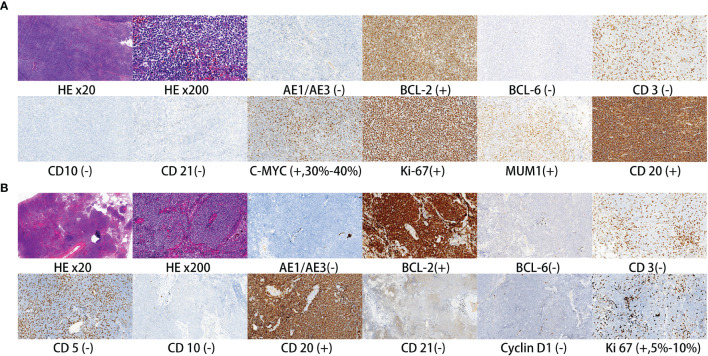
Representative H&E and immunohistochemical staining of pulmonary DLBCL **(A)** and pulmonary MALT **(B)**.

All the 36 patients received surgery besides surgical biopsy. Regarding surgical type, 16 patients received muscle-sparing lung resection (2 wedge resection, 1 segmentectomy, 13 lobectomy). Nineteen patients received video-assisted thoracoscopic surgery (11 wedge resection, 3 segmentectomy, 5 lobectomy). One patient only received thoracoscopic biopsy. Moreover, 22 patients received mediastinal lymphadenectomy, and 4 patients received only biopsy of mediastinal lymph node. Postoperative pathology verified that 8 patients had mediastinal lymph node or hilar lymph node metastasis. The postoperative histopathology showed that there were 31 patients with MALT, 2 with DLBCL, 2 with nodular sclerosing Hodgkin’s lymphoma and 1 with marginal zone B-cell lymphoma. **Figure 2** shows H&E and immunohistochemical staining of pulmonary DLBCL (**Figure 2A**) and pulmonary MALT (**Figure 2B**). Two patients with DLBCL received chemotherapy (rituximab plus CHOP) after surgery. Two patients with nodular sclerosing Hodgkin’s lymphoma received chemoradiotherapy (ABVD plus radiotherapy) after surgery. Five patients (one marginal zone B-cell lymphoma and four MALT) with residual tumor or bilateral pulmonary involvement received local radiotherapy.

**Figure 3:**
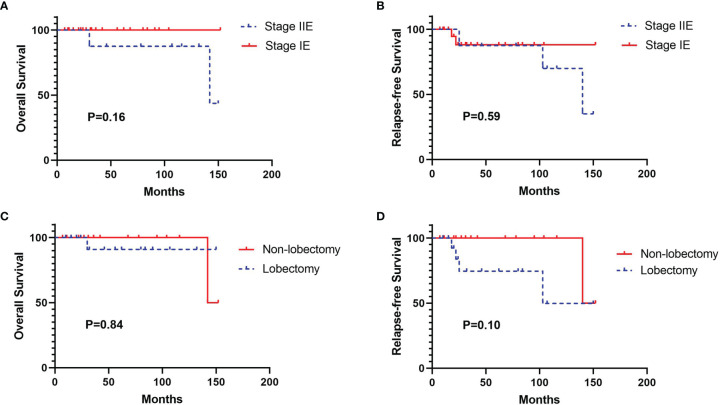
Kaplan-Meier survival curves for overall survival (OS) and relapse-free survival (RFS) according to tumor stage and surgery type. **(A)** OS curve according to tumor stage, **(B)** RFS curve according to tumor stage, **(C)** OS curve according to surgery type, **(D)** RFS curve according to surgery type.

Two patients with MALT were lost to follow-up. The remaining 34 patients had a median follow-up time of 31 months (follow-up range: 7–152 months). During the follow-up period, two patients died and five patients developed progressive disease. One patient with marginal zone B-cell lymphoma (stage II 2E) died of liver metastases, and one patient with MALT (stage II 1E) died of intestinal metastases. The log-rank analysis identified that both tumor stage and surgery type were not associated with OS and RFS (**Figure 3**). There was also no significant predictor found in the univariate and multivariate Cox regression analysis (**Supplementary Tables 1**, **2**).

**Figure 4:**
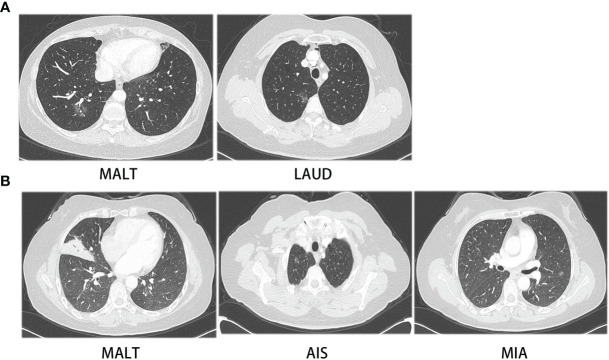
CT images of two patients with synchronous PPL and NSCLC. **(A)** CT images of patient with MALT and LAUD, **(B)** CT images of patient with MALT, AIS and MIA.

Interestingly, two patients were diagnosed with PPL and NSCLC synchronously (**Figure 4**). One patient had a 10- and 17-mm GGO, confirmed to be lung adenocarcinoma and MALT. Both nodules were resected in one surgery. The lung adenocarcinoma had the size of 1 * 0.8 * 0.3 cm. It had high–moderate differentiation and the lepidic subtype predominated with no invasion of pleura, lymph, and nerve. Its stage was IA (T1aN0M0). The other one patient had a 7-mm GGO featuring AIS, a 35-mm consolidation featuring MALT in the right lung, and a 10-mm GGO featuring MIS in the left lung. The patient received VATS wedge resection of the right lung for the first time and received another VATS wedge resection of the left lung after 5 months. As both patients had early-stage lung cancer, regular follow-up after surgery was suggested for them.

Moreover, we divided patients into central tumor and peripheral tumor groups according to tumor location (**Table 2**). Thirty-six patients were classified into 10 central tumors and 26 peripheral tumors. Patients with central tumors had larger tumors than patients with peripheral tumors (*P* = 0.003). Of the 10 patients with central tumors, 60% patients (6 of 10) received lobectomy and 3 patients received palliative resection. Of the 26 patients with peripheral tumors, 61.5% patients (16 of 26) received limited resection, including VATS wedge resection (*n* = 10), muscle-sparing wedge resection (*n* = 2), VATS segmentectomy (*n* = 3), and muscle-sparing segmentectomy (*n* = 1). Patients with peripheral tumors were more prone to receive limited resection, compared with patients with central tumors (*P* = 0.003). For patients with central tumors, 60% of the patients received mediastinal lymph node dissection. For patients with peripheral tumors, 38.5% of the patients received mediastinal lymph node dissection and 19.2% of the patients received lymph node biopsy. Moreover, only two patients had postoperative complications in the group of peripheral tumors. There was no significant difference between these two groups. One patient had arrhythmia and chylothorax after VATS segmentectomy, and another patient had air leak after muscle-sparing lobectomy.

**Table 2:** Clinical characteristics and treatment options in patients with central tumors and peripheral tumors.

Variable	Central tumor (*n* = 10)	Peripheral tumor (*n* = 26)	*P*-value
Age	52 (31–64)	56.5 (38–69)	0.200
Male sex	3 (30.0)	11 (42.3)	0.706
Radiologic findings			
Longest diameter, cm	5.5 (3–10)	2.75 (0.8–13)	0.003
Classification			1.000
Single nodular or consolidation	7 (70.0)	18 (69.2)	
Multiple nodular or consolidation	3 (30.0)	8 (30.8)	
Treatment characteristics			0.003
Lobectomy	6 (60.0)	9 (34.6)	
Limited resection	1 (10.0)	16 (61.5)	
Palliative resection	3 (30.0)	0 (0)	
Biopsy	0 (0)	1 (3.8)	
Mediastinal lymph node dissection	6 (60.0)	10 (38.5)	0.355
Lymph node biopsy	0 (0)	5 (19.2)	
No lymph node dissection	4 (40.0)	11 (42.3)	
Complications	0 (0)	2 (7.7)	1.000

## Discussion

PPL is a rare disease of clonal lymphoid proliferation affecting one or both lungs (14). It has a large difference from lymphoma which is a malignant tumor of the immune system. Most PPLs are indolent with 5-year survival rates of over 85% and median survival of over 10 years in previous studies (13). Regular follow-up is thought to be the proper therapy for most PPL patients. A study from the Sylvester Comprehensive Cancer Center showed that patients with clinically asymptomatic disease followed by observation had no progression during a median follow-up of 40.5 months (15). In this present study, we retrospectively reviewed 36 PPL patients who were first diagnosed at our thoracic department from 2006 to 2020. Most PPLs were MALT which were indolent and associated with favorable outcomes in previous studies. Five PPLs were marginal zone B-cell lymphoma, DLBCL, and HL. Different from previous studies, all the 36 PPL patients received surgery-based modality. We assessed the clinical manifestations, lung lesion features of preoperative examinations, therapy modality, pathology characteristics, and prognosis.

Previous studies showed that gender ratio was controversial. In some literature, males were slightly higher in number than females; however, some Chinese studies got the opposite results (16, 17). In our study, the female to male ratio was 1.57:1. The clinical manifestations of PPL are non-specific. A total of 61.1% of the patients had no clinical symptoms, and 14 other patients had respiratory symptoms like cough, fever, chest distress, chest pain, and bloody sputum. A total of 77.8% (7/9) of patients and 25.9% (7/27) of patients had respiratory symptoms in stage IIE and stage IE, separately. Patients in stage IIE were more prone to have respiratory symptoms than stage IE patients (*P* = 0.014). Twenty-one patients received antibiotics for 1 to 4 weeks before hospitalization. Radiographic images showed no significant change after antibiotic treatment. Consistent with previous studies, our study showed that patients with PPL had favorable outcomes. During the follow-up period, 86% of the patients were in stable condition. Only two patients died of lymphoma progression.

Preoperative examinations like PET-CT, contrast-enhanced CT scan, CT-guided percutaneous lung biopsy, or bronchoscopic biopsy offered helpful information. However, it is still hard to clearly discriminate PPL from lung cancer and pneumonia. PET-CT results suggested that patients with indolent PPL like MALT often had slightly increased SUVmax absorption (from 0 to 6), and the absorption value had no relationship with tumor size. One patient with nodular sclerosing Hodgkin’s lymphoma had a relatively high SUVmax absorption (17.3). Most preoperative biopsy offered the same results with histopathology obtained from surgery. However, due to the small sample size and tumor heterogeneity, preoperative biopsy could not 100% represent the real pathology.

In our study, CT-guided percutaneous lung biopsy and bronchoscopic biopsy got 90% (9/10) and 66.7% (2/3) accuracy rate, separately.

Regarding the CT images, PPL could be divided into four groups. Consistent with previous studies, single or multiple nodules and pneumonia-like with air space consolidation were the main patterns of radiographic features (18, 19). As the clinical features of PPL were poorly defined, most of the patients were initially misdiagnosed as pneumonia, pulmonary tuberculosis, anaerobic bacteria or fungal infections, and lung cancer (9). For the single or multiple solid nodules or masses, PPL should be discriminated from primary lung cancer and metastatic lung cancer. For the pneumonia-like consolidative pattern, PPL should be discriminated from pneumonia, especially in these COVID-19 pandemic days. For the ground-glass opacity, PPL should be discriminated from early-stage lung cancer.

It is conflicting whether PPL should undergo surgical resection or not. Some experts have suggested that surgery brought no survival benefits to PPL patients and it might cause periprocedural complications (20). The aim of surgery for PPL patients should be to preserve lung function. The others consider that surgical resection could suppress PPL progression and prolong complete response, especially for young patients (15, 21). As this study only included patients with lung resection or just surgical biopsy, we could not compare the results of surgical treatment with those of non-surgical treatment. In the future, we would expand the inclusion criteria and include patients with only non-surgical treatment. However, we could get some evidence from the comparison of non-radical resection and radical resection. In the present study, four patients received palliative resection or just surgical biopsy. Among them, one died of tumor progression and one was lost to follow-up. The other 32 patients received radical resection. Among them, 1 was lost to follow-up, 1 patient died of tumor progression, and 3 patients developed progressive disease. Patients with non-radical resection seemed to have a worse survival than patients with radical resection. However, the result should be further verified in a large population. For the peripherally located tumors, restricted lung resection removes tumors and preserves most of the lung function. Moreover, for a large medical center, restricted lung resection like VATS wedge resection or VATS segmentectomy is proficient in manipulating. Periprocedural complications or death could be reduced in low proportion. In our department, only one patient had arrhythmia and chylothorax, and one patient had air leak. No patients died in the perioperative period. In addition, restricted lung resection is also an important method to make a clear diagnosis, because preoperative examinations like PET-CT, contrast-enhanced CT scan, CT-guided percutaneous lung biopsy, or bronchoscopic biopsy cannot get a precise diagnosis. The misdiagnosis could delay the best time of surgery for patients with malignant cancer. Particularly, the surgical option for GGO featuring PPL is hard to make. For those patients, the therapy modality could follow the guidelines of GGO (22). High-risk patients with GGO could be followed up for 3 or 6 months. If the tumor size has no change or it increases, patients are suggested to have a resection. For patients with tumor size less than 6 mm or pure GGO less than 3 cm, routine follow-up may be an appropriate choice. When the preoperative examination cannot discriminate PPL from lung cancer, surgical resection is the appropriate treatment modality.

It is not clear whether lymph node dissection should be performed for PPL. In our department, 60% of the patients received mediastinal lymph node dissection for central tumors and 57.7% of the patients received mediastinal lymph node dissection or lymph node biopsy for peripheral tumors. Of the eight patients with mediastinal lymph node or hilar lymph node metastasis, only three patients had mediastinal or hilar lymph node enlargement in CT images. Lymph node features in the CT images had a poor match with pathology characteristics. Although lymph node dissection can help evaluate tumor stage, there was no significant difference in overall survival rate between patients with stage I or II disease according to previous studies (23). Lymph node dissection may not make a difference in PPL treatment. But for the patient with uncertain diagnosis, intraoperative frozen section examination is indispensable. We suggest that intraoperative frozen section is an effective method to guide resection strategy (24). Only with the precise diagnosis of PPL, lymph node dissection is not mandatory.

Two patients were diagnosed with PPL and NSCLC synchronously. Simultaneous operations were performed in our department. The tumor sizes of synchronous NSCLC were not larger than 1 cm. Preoperative biopsy was hard to perform. For patients with PPL, multiple pulmonary nodules are common. Some of the nodules are PPL of the same origin, but some may be lung cancer of second origin. We recommend to perform restricted lung resection for suspicious nodules of lung cancer.

There were some limitations in our study. Firstly, the study only included 36 patients with PPL confirmed by surgical pathology. The small sample size from one single-center may restrict the spread of the clinical experience. Secondly, the study was a retrospective research. The shortage of retrospective research was inevitable. Thirdly, the surgical modality of the patients did not follow the same strategy. Some patients received mediastinal lymphadenectomy, but some patients only received lymph node biopsy. Lastly, some patients did not have regular follow-up in our hospital. Hence, their information of subsequent treatment may not be credible.

## Conclusion

Our retrospective analysis suggested that most PPLs were indolent. As PPL has no specific clinical and radiological manifestations, the definite diagnosis depends on pathology. Intraoperative frozen section weighs over preoperative biopsy owing to its high accuracy. Wedge resection could be performed for the suspicious nodules at first. The intraoperative frozen section helps to decide whether a more extensive procedure should be performed. For the peripherally located tumors, restricted lung resection like VATS wedge resection or VATS segmentectomy is recommended. For small hospitals with no proficient pathologists, preoperative biopsy should be carefully evaluated. When PPL is hard to discriminate from lung cancer, the multidisciplinary team could make a better therapeutic regimen.

## Data Availability Statement

The raw data supporting the conclusions of this article will be made available by the authors, without undue reservation.

## Author Contributions

YZ contributed to the study concept and design and the critical revision of the manuscript. HS performed the data collection and analysis and drafted the manuscript. All authors contributed to the article and approved the submitted version.

## Funding

This study was supported by the National Natural Science Foundationof China (81902310).

## Conflict of Interest

The authors declare that the research was conducted in the absence of any commercial or financial relationships that could be construed as a potential conflict of interest.

## Publisher’s Note

All claims expressed in this article are solely those of the authors and do not necessarily represent those of their affiliated organizations, or those of the publisher, the editors and the reviewers. Any product that may be evaluated in this article, or claim that may be made by its manufacturer, is not guaranteed or endorsed by the publisher.
