# Supplementary material for: Clinical Features and Surgical Treatment of Primary Pulmonary Lymphoma: A Retrospective Study
Source: Front Oncol. 2022 Feb 3;12:779395. doi: 10.3389/fonc.2022.779395 (PMC8850835; doi:10.3389/fonc.2022.779395)
Supplement: Supplementary file 1 [file Table_1.docx]

Supplementary Table 1. Univariable and multivariable analyses for overall survival (OS)

in patients with primary pulmonary lymphoma.

Supplementary Table 2. Univariable and multivariable analyses for relapse-free survival (RFS) in patients with resected primary pulmonary lymphoma.

| Age |  | Univariable |  |  | Multivariable |  |
| --- | --- | --- | --- | --- | --- | --- |
|  | P | HR | 95% CI | P | HR | 95% CI |
| Age | 0.44 | 109.92 | 0.001-15380819.35 |  |  |  |
| Gender | 0.43 | 141.71 | 0.001-34843628.4 |  |  |  |
| Smoking status | 0.46 | 2.92 | 0.17-48.97 |  |  |  |
| Symptom | 0.44 | 0.008 | 0.000-1420.81 |  |  |  |
| Stage | 0.47 | 0.014 | 0.000-1688.79 |  |  |  |
| Surgery Type | 0.84 | 1.35 | 0.08-22.75 |  |  |  |
| Lymph node dissection | 0.60 | 72.10 | 0.000-7.149E8 |  |  |  |
| Tumor size | 0.45 | 109.01 | 0.001-18625893.05 |  |  |  |

| Age |  | Univariable |  |  | Multivariable |  |
| --- | --- | --- | --- | --- | --- | --- |
|  | P | HR | 95% CI | P | HR | 95% CI |
| Age | 0.34 | 2.42 | 0.40-14.8 |  |  |  |
| Gender | 0.30 | 2.57 | 0.43-15.4 |  |  |  |
| Smoking status | 0.87 | 0.84 | 0.09-7.69 |  |  |  |
| Symptom | 0.08 | 0.12 | 0.01-1.24 |  |  |  |
| Stage | 0.60 | 0.60 | 0.09-3.96 |  |  |  |
| Surgery Type | 0.14 | 5.25 | 0.58-47.47 |  |  |  |
| Lymph node dissection | 0.27 | 105.64 | 0.03-446394.41 |  |  |  |
| Tumor size | 0.27 | 2.90 | 0.44-18.95 |  |  |  |
